# Evaluating the effectiveness of IPTi on malaria using routine health information from sentinel health centres in southern Tanzania

**DOI:** 10.1186/1475-2875-10-41

**Published:** 2011-02-14

**Authors:** Barbara A Willey, Joanna RM Armstrong Schellenberg, Werner Maokola, Kizito Shirima, Mwajuma Chemba, Hassan Mshinda, Pedro Alonso, Marcel Tanner, David Schellenberg

**Affiliations:** 1 Faculty of Infectious and Tropical Diseases, London School of Hygiene and Tropical Medicine, London, UK; 2 Ifakara Health Institute, Dar es Salaam, Tanzania; 3 Barcelona Centre for International Health Research, Barcelona, Spain; 4 Swiss Tropical and Public Health Institute, Basel, Switzerland; 5 University of Basel, Basel, Switzerland

## Abstract

**Background:**

Intermittent preventive treatment of malaria in infants (IPTi) consists of the administration of a treatment dose of sulphadoxine-pyrimethamine (SP) at the time of routine vaccinations. The use of routine Health Management and Information Services (HMIS) data to investigate the effect of IPTi on malaria, anaemia, and all-cause attendance in children aged 2-11 months presenting to 11 health centres in southern Tanzania is described.

**Methods:**

Clinical diagnosis of malaria was confirmed with a positive blood slide reading from a quality assurance laboratory. Anaemia was defined using two thresholds (mild [Hb < 11 g/dL], severe [Hb < 8 g/dL]). Incidence rates between IPTi and non-implementing health centres were calculated using Poisson regression, and all statistical testing was based on the t test due to the clustered nature of the data.

**Results:**

Seventy two per cent of infants presenting in intervention areas received at least one dose of IPTi- 22% received all three. During March 2006 - April 2007, the incidence of all cause attendance was two attendances per person, per year (pppy), including 0.2 episodes pppy of malaria, 0.7 episodes of mild and 0.13 episodes of severe anaemia. Point estimates for the effect of IPTi on malaria varied between 18% and 52%, depending on the scope of the analysis, although adjustment for clustering rendered these not statistically significant.

**Conclusions:**

The point estimate of the effect of IPTi on malaria is consistent with that from a large pooled analysis of randomized control trials. As such, it is plausible that the difference seen in health centre data is due to IPTi, even thought the effect did not reach statistical significance. Findings draw attention to the challenges of robust inference of effects of interventions based on routine health centre data. Analysis of routine health information can reassure that interventions are being made available and having desired effects, but unanticipated effects should trigger data collection from representative samples of the target population.

## Background

The contribution of *Plasmodium falciparum*-caused malaria to mortality and morbidity of African infants and young children remains substantial [1]. Malaria control strategies throughout the African continent have seen considerable increases in financial and human resources investment from governments, non-governmental agencies, and funders alike [1]. The monitoring and evaluation of malaria control programmes is essential, and recently more emphasis has been placed on both by the WHO's Global Malaria Programme and by the Roll Back Malaria (RBM) partnership. RBM's Monitoring and Evaluation Reference Group (MERG) recommends a number of data collection methods to monitor and evaluate malaria control strategies, and highlights as currently under-exploited, the use of routine health management and information system, particularly from randomly-selected malaria sentinel sites [2,3].

Intermittent preventive treatment of malaria in infants (IPTi) consists of the administration of a treatment dose of sulphadoxine-pyrimethamine (SP) at the time of routine vaccinations in the first year of life [4]. In a pooled analysis of six randomized controlled trials (RCTs) of IPTi using SP, the incidence of clinical malaria in the first year of life was reduced by 30% [5], and IPTi was considered a safe and effective anti-malarial strategy, likely to form a useful component of malaria control programmes in relevant settings [6]. In addition to RCTs [5,7-12], studies addressing questions of cost [13,14], safety, drug resistance [15], acceptability [16,17], and the implementation of IPTi through the Expanded Programme on Immunization (EPI) [18] have also been carried out, under the umbrella of the IPTi consortium [19]. The community effectiveness of IPTi and its impact on infant health has been investigated in Tanzania through a cluster-randomized controlled trial including representative pre and post-intervention household surveys [20,21].

In this paper, data from a consolidated HMIS in 11 sentinel health centres in rural southern Tanzania are used to report (i) the uptake of IPTi and coverage of corresponding EPI vaccines, (ii) the incidence of malaria, anaemia, and all-cause attendance at the health centre, and (iii) the effectiveness of IPTi on the incidence of these outcomes in health centre attendees aged 2-11 months.

## Methods

### Study area and the IPTi strategy

**Figure 1:**
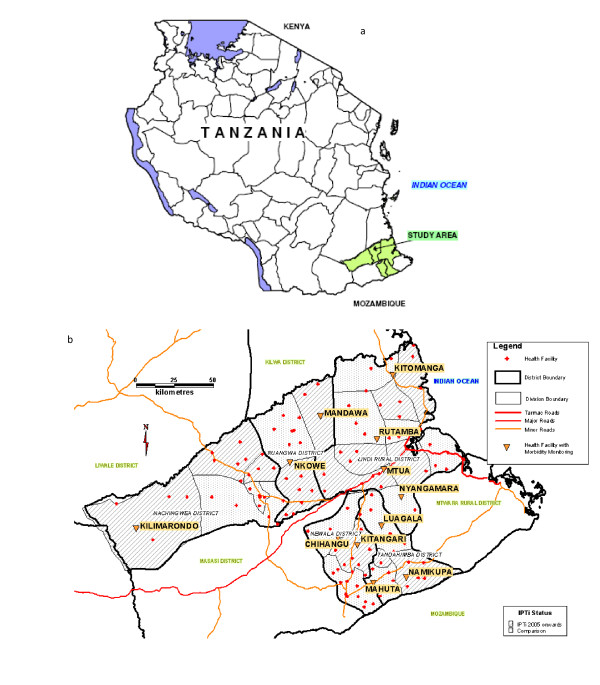
**Map of the study area within Tanzania (a), and the 11 divisions included in the Morbidity Monitoring study (b)**. Note: In map (b) IPTi intervention divisions are shown with diagonal shading, comparison divisions with dotted shading, and Morbidity Monitoring sentinel health centres by orange triangles.

The area has been previously described, but briefly malaria is endemic, with transmission occurring throughout the year, and is the most common primary cause of health centre or hospital admission among those aged under five years [20,22]. The first-line anti-malarial drug in this area was SP, until December 2006, when it changed to artemether-lumefantrine. During the study period a number of malaria control interventions were implemented. Nationwide, from May 2006, the Tanzania National Voucher Scheme was in effect, delivering bed nets bundled with insecticide re-treatment kits to pregnant women through vouchers available at antenatal care clinics [23]. Health centres nationwide also distributed free insecticide re-treatment kits to infants when they attended government clinics for measles vaccination. Furthermore, in Lindi region (Figure 1) during August 2005, bed nets bundled with insecticide were distributed to children aged less than five years during an integrated mass measles vaccination, helminth treatment, and vitamin A supplementation campaign [24].

This study, termed the IPTi Morbidity Monitoring study, took place in the context of a large cluster randomized trial that was designed to determine the community effectiveness of IPTi with SP on malaria and anaemia. IPTi using SP was introduced to 12 of 24 randomly selected divisions, located within five districts, and two regions (Lindi and Mtwara) of rural southern Tanzania (Figure 1) [22]. IPTi effectiveness was estimated through representative household surveys that took place in all 24 participating divisions at baseline in 2004 (n = 21,600), and post IPTi in July/August 2006 (n = 5,760) [20]. IPTi, in the form of a single dose of SP, was delivered in intervention divisions through existing government health centres when children presented for their routine EPI vaccine doses of DPT2, DPT3, and measles (given at two, three, and nine months of age, respectively). Children presenting at government health centres in comparison divisions received their routine EPI vaccine, but no IPTi.

### The IPTi Morbidity Monitoring study, data collection and outcome definition

The Morbidity Monitoring study aimed to monitor the delivery of IPTi with SP through routine contacts with the health service, and to enable evaluation of the effectiveness of IPTi using routine health centre data. All 11 health centres with functional microscopes in the study area were included. These were located in 11 of the 24 divisions included in the IPTi trial, six in intervention, and five in comparison areas (Figure 1, Table 1). On the basis of routine data available for 2004, intervention and comparison areas for this study were comparable. The total catchment population was 68,938 in comparison areas, compared to 69,042 in IPTi-intervention areas. Analysis of routine health centre data suggested that overall malaria incidence was similar in intervention (1.05 episodes per child per year) and comparison areas (1.10 episodes per child per year), and measles vaccination coverage was also similar (1,647 doses in comparison versus 1,594 doses in intervention areas). Implementation of the Morbidity Monitoring was staggered, beginning in 2005, but a period of maximal functionality was defined as the 12 months between April 2006 and March 2007, and results in this paper refer to this period.

**Table 1:** Distribution of children by health centre, characteristics of the Morbidity Monitoring study population, and uptake of EPI and IPTi doses

	Intervention	Comparison	p value *
**Number children per health centre [n, (%)]**	n = 5035	n = 4845	
A	486 (4.9)		
B	869 (8.9)		
C	862 (8.7)		
D	999 (10.1)		
E	951 (9.6)		
F	868 (8.8)		
G		840 (8.5)	
H		519 (5.2)	
I		1198 (12.1)	
J		1610 (16.3)	
K		678 (6.9)	

**Demographic characteristics**			
Sex [n/N, (%)]			
Male	2567/5035 (51)	2514/4845 (52)	0.525

Birth order [n/N, (%)]			
Singleton	4912/5035(98)	4685/4845 (97)	0.533

Median age at entry into analysis [months, (IQR)]	4 m 3 d (2 m-7 m 24 d)	4 m 12 d (2 m-7 m 27 d)	0.742

**Health centre attendance**			
≥1 health centre attendance [n/N, (%)]	2160/5035 (43)	2067/4845 (43)	0.799
Median number of attendances (IQR)	2 (1-3)	2 (1-3)	0.892

**EPI vaccine uptake [n/N, (%)]**			
DPT dose 2	3618/5035(72)	3181/4845 (66)	0.204
DPT dose 3	2889/4794 (60)	2296/4662 (49)	0.019
Measles vaccine	1609/3208 (50)	1009/3065 (33)	0.003

**Median age at EPI vaccinations [months, (IQR)]**			
DPT dose 2	2 m 18 d(2 m 6 d-3 m 9 d)	2 m 24 d (2 m 9 d-3 m 21 d)	0.435
DPT dose 3	4 m 0 d(3 m 9 d-5 m 0 d)	4 m 3 d (3 m 12 d-5 m 9 d)	0.585
Measles vaccination	9 m 18 d (9 m 6 d-10 m 15 d)	9 m 15 d (9 m 3 d-10 m6 d)	0.099

**IPTi uptake [n/N, (%)]**			
IPTi (at least one dose)	3627/5035 (72)		
IPTi (all three doses)	1133/5035 (22)		

**Median age at IPTi doses [months, (IQR)]**			
IPTi dose 1	2 m 15 d (2 m 3 d-3 m 6 d)		
IPTi dose 2	4 m 0 d (3 m 9 d-5 m 0 d)		
IPTi dose 3	9 m 18 d (9 m 3 d-10 m 12 d)		

* Using unpaired t test p value and approximate 95% CI that adjust for clustering by health centre

EPI = expanded programme on immunization, IQR = inter-quartile range, m = months, d = days

Routine HMIS data, including demographic information, Outpatient department attendance (OPD) and vaccination records were captured electronically by study-trained local school leavers, using Personal Digital Assistants (PDAs) (Palm m130) [25], and records were linked using an identification code. Each PDA operator was working full-time in one of the 11 health centres. They were supported by the study's data entry clerk who visited each health centre monthly.

In addition to routine HMIS data, blood slide readings for malaria parasites and haemoglobin data were also collected. Malaria parasite blood slides were prepared using Giemsa staining, and parasite counts per 200 white blood cells (WBCs) were calculated [26,27], while haemoglobin was measured using the Haemoglobin Colour Scale [28]. Following reading at the health centre, malaria parasite blood slides were stored and read twice in the Morbidity Monitoring study's quality assurance (QA) laboratory by two independent slide readers [26,29]. Malaria in this study was defined as a health centre attendance which resulted in both a recorded malaria diagnosis by the health centre clinician, and a positive blood slide reading (≥1 parasite/200 WBCs) from the QA laboratory. Health centre blood slide results were not used to define malaria, as these had an average sensitivity of 89.6% and specificity of 77.3%, using the quality assurance laboratory as the gold standard, and variability between health centres was seen. Anaemia was defined using two thresholds; mild anaemia was a haemoglobin (Hb) concentration below 11 g/dL, and severe anaemia used a threshold of Hb < 8 g/dL.

### Statistical methods

Data from PDAs were exported using Pendragon Forms software to Microsoft Access for data management, and subsequently to Stata 10.0 for statistical analysis (Stata Corp., College Station, TX, USA). Analysis was done according to a pre-agreed analytical plan, incorporating both 'intention to treat' and 'per protocol' analyses. In 'intention to treat' analyses, all children attending a sentinel health centre located in divisions randomized to receive IPTi were compared with those attending sentinel health centres located in IPTi comparison divisions. For the 'per protocol' analyses, children attending health centres in intervention areas who had received IPTi were compared with those attending health centres in comparison areas who had received the corresponding EPI vaccines.

Time at risk for malaria, anaemia, and OPD incidence rate calculations began at the latest of the following: date of registration in the consolidated HMIS, date of becoming two months old, or start date of the study (1^st ^April 2006), and ended at the earliest of either becoming 12 months of age, or the end date of the study (31^st ^March 2007). In order to avoid double-counting the same malaria episode as two separate episodes, 28 days follow up was removed after each malaria diagnosis in analyses of multiple episodes of malaria. Incidence rates were calculated using Poisson regression, and incidence rates of multiple episodes of malaria or all-cause health centre attendance were adjusted for within-child clustering using random effects Poisson regression modelling. The protective efficacy of IPTi was defined as (1-RR) × 100, where RR is the rate ratio. All statistical testing was based on the t test, using a summary measure of the data from each health centre, to take into consideration the clustered nature of the health centre data [30].

In addition to analyses specified in the pre-agreed analytical plan, prevalence of malaria and anaemia in infants using data from the 2004 and 2006 IPTi community effectiveness household surveys was also calculated. These surveys are described in detail elsewhere [20,22], but were large, representative of households in the 24 divisions included in the IPTi cluster randomized controlled trial, and the 2006 survey took place in July and August, overlapping the time period of this study. Results of these secondary analyses presented here are restricted to the 11 divisions that were also included in the IPTi Morbidity Monitoring study. Statistical testing was based on cluster level t tests, and analyses were carried out using *svy *commands in Stata, which are methods appropriate for the clustered design of the IPTi trial and the sampling strategy of the household surveys [30].

### Ethics

The study was undertaken within the framework of the assessment of the community effectiveness of IPTi, part of the IPTi Consortium. Ethical approval was granted by the following local and national institutional review boards: Ifakara Health Institute, National Tanzania Medical Research Coordinating Committee, London School of Hygiene & Tropical Medicine, and the Cantons of Basel-Stadt and Basel-Land, Switzerland. The permission to execute the project was sought and obtained from the Tanzania Ministry of Health and Social Welfare and the respective District Councils of the participating districts.

## Results

### Characteristics of the Morbidity Monitoring study population

A total of 9,880 children aged 2-11 months contributed at least one day to time at risk between April 2006 and March 2007. Half were from health centres in intervention areas, although some health centres saw more children than others (Table 1). Median registration date was 27^th ^April 2006, and median age at entry into the analysis (four months) was similar in intervention and comparison areas (Table 1). In total, therefore, 7,056 health centre visits were recorded in 4,227 children, with a median of two all-cause health centre attendances per child in both intervention and comparison areas (Table 1).

### Uptake of EPI vaccinations and IPTi doses

During the study, uptake of routine EPI vaccinations was 69% for DPT2 (children aged 2-11 months), 55% for DPT3 (children aged 3-11 months), and 42% for measles (children aged 9-11 months). Similar proportions of children from both areas received DPT2; while more children from the intervention areas received DPT3 and measles vaccines (Table 1). Median age at DPT2, DPT3 and measles was 2 months 21 days, 4 months 0 days, and 9 months 18 days respectively, and median age at these routine vaccinations was similar between children in intervention and comparison areas (Table 1). A total of 72% of children aged 2-11 months in intervention areas received at least one dose of IPTi, and 22% received all three doses. Median age at IPTi1, IPTi2 and IPTi3 was 2 months 15 days, 4 months 0 days, and 9 months 18 days respectively (Table 1).

### IPTi and malaria, anaemia, and all-cause OPD attendance

Point estimates suggest a protective effect of IPTi on malaria, but an increase in the risk of anaemia, and no difference in the incidence of all-cause outpatient attendance. However, adjustment for the clustered nature of the data means that none of these results reached statistical significance (Tables 2, 3, and 4).

**Table 2:** Incidence of first or only and all episodes of malaria, and incidence in all time at risk after IPTi dose, and within 28 days of IPTi dose one

	Intervention Area			Comparison Area			Effect		
Intention to treat *	Events	PYAR	Rate	Events	PYAR	Rate	RR (95% CI)	p value	PE (95% CI)

**Malaria incidence (first or only)**									
*Crude*	315	2048.3	0.154	448	1882.7	0.238	0.65 (0.56-0.75)	< 0.001	35% (25%,44%)
*adjusted *^‡^							0.75 (0.24-2.31)	0.584	25% (-131%,76%)

**Malaria incidence (all episodes)**									
*Crude*	329	2105.9	0.156	507	1946.0	0.261	0.60 (0.52-0.69)	< 0.001	40% (31%,48%)
*adjusted *^† ^^‡^							0.82 (0.29-2.29)	0.663	18% (-129%,71%)

	IPTi Intervention Area			Corresponding EPI vaccine Comparison Area			Effect		
Per protocol **	Events	PYAR	Rate	Events	PYAR	Rate	RR (95% CI)	p value	PE (95% CI)

**Malaria incidence (all episodes) in ATAR since IPTi dose**									
IPTi dose 1									
*crude*	79	623.2	0.127	125	513.1	0.244	0.52 (0.39-0.69)	< 0.001	48% (31%,61%)
*adjusted *^† ‡^							0.68 (0.21-2.22)	0.498	32% (-122%,79%)

**Malaria incidence within 28 days of IPTi dose**									
IPTi dose 1									
*crude*	7	95.1	0.070	13	81.0	0.160	0.46 (0.18-1.15)	0.088	54% (-15%,82%)
*adjusted *^‡^							0.48 (0.11-2.13)	0.341	52% (-113%,89%)

* Intention to treat analyses comparing IPTi intervention and comparison areas

** Per protocol analyses comparing children who received IPTi in intervention areas to those that received corresponding EPI vaccines in comparison areas

EPI = expanded programme on immunization, PAYR = person years at risk, ATAR = all time at risk, RR = rate ratio, CI = confidence interval, PE = protective efficacy

^† ^Results adjusted for clustering within children for multiple episodes of malaria using Poisson random effects modeling

^‡ ^Using intervention and comparison area mean rate, unpaired t test p value and approximate 95% CI that adjust for clustering by health centre

**Table 3:** Incidence of first or only episode of mild and severe anaemia, and incidence of anaemia in all time at risk, and within 28 days of IPTi dose one

	Intervention Area			Comparison Area			Effect		
Intention to treat *	Events	PYAR	Rate	Events	PYAR	Rate	RR (95% CI)	p value	PE (95% CI)

**Mild anaemia incidence (first or only)**									
*Crude*	1268	1771.8	0.716	1170	1689.7	0.694	1.03 (0.95-1.12)	0.424	-3% (-12%,5%)
*adjusted *^‡^							1.13 (0.65-1.97)	0.597	-13% (-97%,35%)

**Severe anaemia incidence (first or only)**									
*crude*	318	2046.8	0.155	206	1944.1	0.106	1.47 (1.23-1.75)	< 0.001	-47% (-75%,-23%)
*adjusted *^‡^							1.59 (0.54-4.68)	0.360	-59% (-368%,46%)

	IPTi Intervention Area			Corresponding EPI vaccine Comparison Area			Effect		

Per protocol **	Events	PYAR	Rate	Events	PYAR	Rate	RR (95% CI)	p value	PE (95% CI)

**Mild anaemia incidence (first or only) in ATAR since IPTi dose**									
IPTi dose 1									
*crude*	289	543.8	0.531	257	455.6	0.564	0.93 (0.78-1.10)	0.383	7% (-10%,22%)
*adjusted *^‡^							1.04 (0.58-1.87)	0.878	-4% (-87%,42%)

**Severe anaemia incidence (first or only) in ATAR since IPTi dose**									
IPTi dose 1									
*crude*	65	615.2	0.106	26	518.3	0.050	2.10 (1.34-3.32)	0.001	-110% (-232%,-34%)
*adjusted *^‡^							2.12 (0.41-11.03)	0.277	-112% (-1000%,59%)

**Mild anaemia incidence (first or only) within 28 days of IPTi dose**									
IPTi dose 1									
*crude*	45	93.8	0.480	31	80.5	0.385	1.25 (0.79-1.97)	0.343	-25% (-97%,21%)
*adjusted *^‡^							1.30 (0.42-4.07)	0.579	-30% (-307%,58%)

**Severe anaemia incidence (first or only) within 28 days of IPTi dose**									
IPTi dose 1									
*crude*	4	95.1	0.042	2	81.4	0.025	1.71 (0.31-9.35)	0.530	-71% (-835%,69%)
*adjusted *^‡^							1.57 (0.11-22.93)	0.700	-57% (-2190%,89%)

* Intention to treat analyses comparing IPTi intervention and comparison areas

** Per protocol analyses comparing children who received IPTi in intervention areas to those that received corresponding EPI vaccines in comparison areas

EPI = expanded programme on immunization, PAYR = person years at risk, ATAR = all time at risk, RR = rate ratio,

CI = confidence interval, PE = protective efficacy,

Hb = haemoglobin

^‡ ^Using intervention and comparison area mean rate, unpaired t test p value and approximate 95% CI that adjust for clustering by health centre

**Table 4:** Incidence of first or only and all OPD attendances, and incidence of attendance in all time at risk, and within 28 days of IPTi dose one

	Intervention Area			Comparison Area			Effect		
Intention to treat *	Events	PYAR	Rate	Events	PYAR	Rate	RR (95% CI)	p value	PE (95% CI)

**Health centre attendance (first or only)**									
*crude*	2160	1535.3	1.407	2067	1434.4	1.441	0.97 (0.92-1.04)	0.417	3% (-4%,8%)
*adjusted *^‡^							1.04 (0.75-1.45)	0.795	-4% (-45%,25%)

**Health centre attendances (all episodes)**									
*crude*	3631	1810.5	2.005	3425	1699.3	2.015	0.99 (0.95-1.04)	0.834	1% (-4%,5%)
*adjusted *^† ^^‡^							1.05 (0.75-1.48)	0.736	-5% (-48%,25%)

	IPTi Intervention Area			Corresponding EPI vaccine Comparison Area			Effect		

Per protocol **	Events	PYAR	Rate	Events	PYAR	Rate	RR (95% CI)	p value	PE (95% CI)

**Health centre attendances (all episodes) in ATAR since IPTi dose**									
IPTi dose 1									
*crude*	1109	565.2	1.960	1082	466.6	2.317	0.84 (0.78-0.92)	0.001	16% (8%,22%)
*adjusted *^† ^^‡^							0.98 (0.69-1.38)	0.907	2% (-38%,31%)

**Health centre attendance (all episodes) within 28 days of IPTi dose**									
IPTi dose 1									
*crude*	106	93.7	1.131	125	80.3	1.556	0.73 (0.56-0.94)	0.016	27% (6%,44%)
*adjusted *^† ^^‡^							0.82 (0.48-1.40)	0.421	18% (-40%,52%)

* Intention to treat analyses comparing IPTi intervention and comparison areas

** Per protocol analyses comparing children who received IPTi in intervention areas to those that received corresponding EPI vaccines in comparison areas

EPI = expanded programme on immunization, PAYR = person years at risk, ATAR = all time at risk, RR = rate ratio, CI = confidence interval, PE = protective efficacy

^† ^Results adjusted for clustering within children for multiple episodes of all-cause health centre attendance using Poisson random effects modeling

^‡ ^Using intervention and comparison area mean rate, unpaired t test p value and approximate 95% CI that adjust for clustering by health centre

### Malaria

In total during this 12 month period, 763 first or only episodes and 836 multiple episodes of malaria were seen (Table 2). Rates of malaria in IPTi intervention areas appeared lower in 'intention to treat' analysis comparing children attending health centres in IPTi intervention and comparison areas, although differences did not reach statistical significance (RR = 0.75, 95% CI 0.24-2.31 first or only episode of malaria; RR = 0.82, 95% CI 0.29-2.29 multiple episodes). In 'per protocol' analyses, comparing children from intervention areas who received IPTi dose 1 with children from comparison areas who received DPT2, 204 episodes of malaria were seen in all time at risk after receiving IPTi dose 1, and 20 episodes were recorded within 28 days of IPTi dose 1. Rates of malaria in children who received IPTi 1 appeared lower, but differences were not statistically significant (RR = 0.68, 95% CI 0.21-2.22 for episodes in all time at risk after IPTi 1; RR = 0.48, 95% CI 0.11-2.13 for episodes with 28 days). Protective efficacy point estimates in both 'intention to treat' and 'per protocol' analyses were consistently lower on adjustment for clustering by health centre.

### Anaemia

Table 3 shows that 2438 episodes of mild anaemia (Hb < 11 g/dL), and 524 episodes of severe anaemia (Hb < 8 g/dL) were recorded during the 12 month study period. Point estimates of protective efficacy in 'intention to treat' analyses suggest rates in children attending health centres in IPTi intervention areas were higher that those attending centres in comparison areas, although differences were not statistically significant (RR = 1.13, 95% CI 0.65-1.97 mild anaemia; RR = 1.59, 95% CI 0.54-4.68 severe anaemia). In 'per protocol' analyses 546 episodes of mild anaemia were seen in all time at risk (ATAR) since IPTi dose 1, and 76 episodes were recorded within 28 days. Rates of severe anaemia were much lower, with 91 in all time at risk after IPTi dose 1, and only six cases of severe anaemia within 28 days of IPTi dose 1. Rates of mild and severe anaemia from 'per protocol' analyses were also higher in children who had received IPTi dose 1, in comparison to those who had received DPT2, but results were not statistically significant (mild anaemia: RR = 1.04, 95% CI 0.58-1.87 ATAR and RR = 1.30, 95% CI 0.42-4.07 within 28 days; severe anaemia: RR = 2.12, 95% CI 0.41-11.03 ATAR, and RR = 1.57, 95% CI (0.11-22.93) within 28 days).

### All-cause attendance at the health centre

Over the 12 months from April 2006 to March 2007, 4,227 first or only attendances, and 7,056 multiple attendances at the health centre were seen (Table 4). Rates of attendance in children visiting health centres in IPTi intervention areas were similar to those in children attending centres in comparison areas. In 'per protocol' analyses 2191 attendances were recorded in all time at risk after IPTi dose 1, and 231 attendances were recorded within 28 days of IPTi 1. Rates of all-cause health centre attendance were similar in children from IPTi intervention areas who received IPTi dose 1 than in those from comparison areas who had received DPT2.

### Malaria and anaemia prevalence in the 2004 and 2006 IPTi household surveys, restricted to Morbidity Monitoring divisions

**Figure 2:**
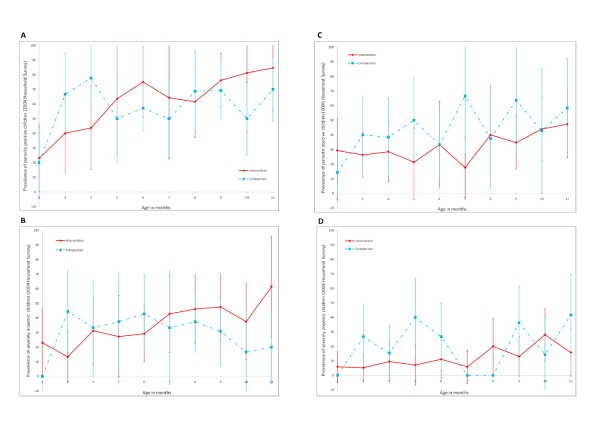
**Prevalence of parasitaemia and severe anaemia (Hb < 8 g/dL) with 95% confidence intervals, by age in months, in children aged 2-11 months in Morbidity Monitoring divisions (n = 11). Data from 2004 and 2006 IPTi Household Surveys**. (A) Prevalence of parasite positive children (2004 Household Survey), n = 257. (B) Prevalence of severely anaemic children (2004 Household Survey), n = 257. (C) Prevalence of parasite positive children (2006 Household Survey), n = 299. (D) Prevalence of severely anaemic children (2006 Household Survey), n = 299. Hb = haemoglobin

When restricted to the 11 divisions included in the Morbidity Monitoring study, information was available from 257 and 319 children aged 2-11 months in the 2004 and 2006 household surveys respectively. Results from the 2004 survey (prior to IPTi), suggest that the 11 divisions included in the IPTi Morbidity Monitoring study were comparable on variables for which information was available (Table 5). Changes between 2004 and 2006 in the prevalence of parasitaemia, severe anaemia and net use by children in the night before the survey (Figure 2, Table 5), show a marked decrease in malaria parasitaemia, and an increase in mean haemoglobin and bed net usage in both IPTi intervention and comparison areas. In 2006, in Morbidity Monitoring areas, differences between intervention and comparison areas did not reach statistical significance, but prevalence of parasitaemia was nine percentage points lower in intervention areas, while mean haemoglobin was 0.3 g/dL higher in intervention areas, and prevalence of reported bed net use by children in the night before the survey was 14 percentage points higher in comparison areas (Table 5).

**Table 5:** Prevalence of parasitaemia, net use and mean haemoglobin levels in children aged 2-11 months, using data from the 2004 and 2006 IPTi Household Surveys, restricted to Morbidity Monitoring divisions (n = 11)

2004 IPTi household survey	Intervention	Comparison	p value *
Parasitaemia [*n/N (%)*]	98/156 (63)	60/101 (59)	0.677
Haemoglobin (g/dL) [*mean (95% CI)]*	8.5 (8.3-8.8)	8.8 (8.5-9.1)	0.955
Net use (last night) [*n/N (%)*]	45/156 (29)	27/101 (27)	0.739

**2006 IPTi household survey**	**Intervention**	**Comparison**	p value *

Parasitaemia [*n/N (%)*]	62/188 (33)	47/111 (42)	0.130
Haemoglobin (g/dL) [*mean (95% CI)*]	9.6 (9.3-9.9)	9.3 (8.9-9.6)	0.640
Net use (last night) [*n/N (%)*]	102/195 (52)	77/116 (66)	0.519

Results obtained using *svy *commands in Stata, which adjust for the sampling strategy of the survey

Hb = haemoglobin, CI = confidence interval

* Using unpaired t test that adjusts for the IPTi trial study design (randomized by division)

## Discussion

### Effectiveness of IPTi with SP

In all, 72% of children aged 2-11 months attending health centres in intervention areas received at least one dose of IPTi, and 22% received all three doses. Median age at IPTi dose was older than recommended by EPI guidelines, but similar to median ages at routine vaccinations, and similar to those seen during the 2006 IPTi representative household survey [22]. Results suggest that more children from intervention areas received DPT2 and measles vaccine, however, results from the representative 2006 IPTi household survey, carried out during a time period that overlapped this study, show no indication of increased levels of EPI coverage in IPTi intervention areas-coverage of DPT vaccination was identical in both areas at 74% of children aged 6-11 months [22].

Overall incidence rates of 0.2 episodes of lab-confirmed malaria; 0.7 episodes of mild and 0.13 of severe anaemia; and 2 episodes of all-cause health centre attendance per child per year were seen. These analyses of routine health information data suggest that IPTi was associated with an increased risk of anaemia, a reduced risk of malaria, an increase in coverage of routine vaccinations, and no effect on all cause out-patient attendance. However, none of the effects of IPTi on malaria, anaemia or all-cause health centre attendance, in either 'intention to treat' or 'per protocol' analyses, reached statistical significance when adjusted for clustering by health centre. The confidence intervals for the rate ratios of the effect of IPTi with SP on these outcomes included the null value of one, and ranged from rate ratios that are compatible with a protective effect of IPTi, to rate ratios that suggest increased rates of these outcomes in the IPTi group. These results could represent (i) that IPTi with SP has no impact on malaria, anaemia or all-cause attendance at the health centre in children aged 2-11 months in this setting, (ii) there was insufficient statistical power to detect an effect of IPTi with SP, and linked to this, (iii) that the background rates of malaria and malaria-related anaemia were too low, due possibly to other control measures including net use, to detect an impact of IPTi with the existing study design.

Studies, including a pooled analysis of six large RCTs of IPTi using SP [5], have reported an IPTi-related reduction in malaria and health centre attendance in infants. Increased rates of anaemia have been reported in two IPTi trials [9,11], however, the majority of RCTs of IPTi have reported a decline [7,8,12], or no effect [10], with results of the pooled analysis showing a 22% decline in severe anaemia (Hb < 8 g/dL) [5]. Results from the 2006 IPTi household survey carried out in July and August, and including all 24 divisions, indicated that prevalence of mild anaemia (Hb < 11 g/dL) was 8% lower (p = 0.02) in children from IPTi intervention areas, while in children who had received IPTi, mean haemoglobin was 0.27 grams higher (p = 0.05) than in children who had received the corresponding EPI vaccine [22]. Given findings from the wider literature, it is suggested that the discrepancy in the results seen in this study, and those from the IPTi household survey carried out during an overlapping time period in the same geographical area, is more likely due to the non-representative nature of infants attending health facilities in comparison to the whole population of infants, rather than to a true increased risk in anaemia associated with IPTi using SP.

All health centres with functional microscopes in our project area were included in this evaluation (n = 11). However, this provided only 32% power to detect a 30% difference between IPTi intervention and comparison areas. An additional 19 centres (30 in total) would be required to achieve 80% power [30], although this would not have been feasible, as in reality all eligible centres in participating divisions were included.

In the 11 divisions included in the Morbidity Monitoring study, there was a 17% fall in malaria parasitaemia between 2004 and 2006 in children aged 2-11 months of age living in IPTi comparison areas, as measured by the representative household surveys. Using the same source data, it was shown that in children of this age group in IPTi comparison areas there was also a 0.5 g/dL increase in mean haemoglobin, and a 39% increase in the reported use of nets by children on the night before the survey. These background changes may also have contributed to the current study lacking sufficient power to detect an effect of IPTi with SP. Longer periods of observation may enable the discernment of trends, and a difference between these trends in intervention and comparison areas. These observations also draw attention to the need to monitor contextual factors which may impact on the evaluation.

### The use of routine HMIS data to monitor and evaluate malaria control programmes

This study used HMIS data to evaluate the effectiveness of IPTi with SP on health centre attendees. Findings may, therefore, not be representative of children, and their morbidity burden, in the wider community [31].

The quality of data in HMIS remains a critical issue. Monthly supervision visits to all study sites were carried out by the study co-coordinator (WM), data manager (KS), and laboratory assistant. Visits were an opportunity to discuss issues of data completeness and quality with health centre staff. The basis for these discussions was an automatically-, PDA-generated monthly report. These compared numbers of OPD attendances and EPI vaccinations to those recorded during the previous month; highlighted whether all OPD attendances had corresponding laboratory records; and listed the top ten diagnoses made at the centre in the past month, which previously had been prepared manually. Use of PDAs enhanced data capture, ease of retrieval and summarising of the information. However, despite monthly supervision, issues of data completeness across HMIS registers, and the quality of microscopy from the on-site health centre laboratories remained.

For example, on average 41% of blood slide results from the health centre laboratory were missing a corresponding record in the OPD register, and this showed variability between the different health centres. This study used blood slide results from the Morbidity Monitoring quality assurance laboratory, along with clinical diagnosis from HMIS, to classify malaria outcome. Health centre microscopy results had an average sensitivity of 89.6% and specificity of 77.3%, using the QA lab as gold standard. This again showed variation between health centres. There was no suggestion that data completeness or quality of microscopy was consistently better or worse in IPTi intervention or comparison areas.

The use of HMIS data for monitoring and evaluation should undergo quality control checks, be analysed with due regard to potential selection biases (e.g. the type of patient attending different health facilities), clustering of the data, and statistical power considerations. The data need to be interpreted bearing in mind the contextual situation and consistency of results. Where unexpected findings are documented, well-designed, well-implemented, representative household surveys may be needed to answer the question of intervention effectiveness at the community level, also capturing data from those populations who do not attend routine health services.

## Conclusions

This study used routine data from Health Management Information Systems in 11 sentinel health centres in rural southern Tanzania to evaluate the effectiveness of a new malaria control strategy, IPTi with SP. The process highlights the challenges of using routine health centre data to monitor and evaluate the effectiveness of a malaria control programme in health centre attendees. Findings suggest that unexpected results should be investigated by drawing representative samples of the target population to enable robust conclusions to be drawn.

## Competing interests

The authors declare that they have no competing interests.

## Authors' contributions

The paper was drafted by BW, WM, JAS and DS. BW carried out the statistical analyses, with support from JAS. DS, JAS, HM, PA and MT participated in the set up and management of the Morbidity Monitoring study. WM, KS and MC were responsible for data collection. All authors commented on drafts of the paper and approved the final version.
